# Monitoring sleep using smartphone data in a population of college students

**DOI:** 10.1038/s44184-023-00023-0

**Published:** 2023-03-17

**Authors:** Carsten Langholm, Andrew Jin Soo Byun, Janet Mullington, John Torous

**Affiliations:** 1 Division of Digital Psychiatry, Beth Israel Deaconess Medical Center, Harvard Medical School, Boston, MA 02215 USA; 2 John A. Paulson School of Engineering and Applied Sciences, Harvard University, Cambridge, MA 02138 USA; 3 Department of Neurology, Beth Israel Deaconess Medical Center, Harvard Medical School, Boston, MA 02215 USA

**Keywords:** Medical research, Translational research

## Abstract

Sleep is fundamental to all health, especially mental health. Monitoring sleep is thus critical to delivering effective healthcare. However, measuring sleep in a scalable way remains a clinical challenge because wearable sleep-monitoring devices are not affordable or accessible to the majority of the population. However, as consumer devices like smartphones become increasingly powerful and accessible in the United States, monitoring sleep using smartphone patterns offers a feasible and scalable alternative to wearable devices. In this study, we analyze the sleep behavior of 67 college students with elevated levels of stress over 28 days. While using the open-source mindLAMP smartphone app to complete daily and weekly sleep and mental health surveys, these participants also passively collected phone sensor data. We used these passive sensor data streams to estimate sleep duration. These sensor-based sleep duration estimates, when averaged for each participant, were correlated with self-reported sleep duration (*r* = 0.83). We later constructed a simple predictive model using both sensor-based sleep duration estimates and surveys as predictor variables. This model demonstrated the ability to predict survey-reported Pittsburgh Sleep Quality Index (PSQI) scores within 1 point. Overall, our results suggest that smartphone-derived sleep duration estimates offer practical results for estimating sleep duration and can also serve useful functions in the process of digital phenotyping.

## Introduction

Sleep remains fundamental to all health. Insufficient sleep duration causes many negative health outcomes, but can be particularly detrimental to mental health by increasing the prevalence and severity of depression and anxiety^1–3^. Therefore, some consider lack of sleep an under-recognized public health epidemic^4^. Properly maintaining mental health, therefore, benefits from monitoring sleep duration and habits. In fact, sleep monitoring often comprises the assessment of therapeutic response to mental health intervention. However, capturing data on sleep duration continues to be challenging.

While patients can complete detailed sleep logs as a means to record sleep habits, these logs can be burdensome and often lead to low levels of engagement. High attrition has driven the search for new solutions. Some of these proposed solutions involve actigraphy, due to its simplicity and accuracy. However, actigraphy devices come at a high monetary cost. On the contrary, cheaper and more prevalent consumer devices like smartphones and wearables offer a more scalable potential. Implementing consumer devices in sleep monitoring can be challenging. As a result, the Sleep Research Society considers smartphone-based sleep monitoring results to be “premature”^5^. The challenge to employing consumer devices to measure sleep habits stems not from a lack of capability in consumer technologies. On the contrary, today’s smartphones offer many impressive sensors capturing high-frequency data relevant to sleep, such as motion, location, ambient light, screen time, and even LIDAR (laser imaging, detection, and ranging)^6^. The Sleep Research Society instead believes the primary challenge to monitoring sleep habits using smartphones arises from use and interest outpacing research and validation^5^.

While there has been a recent focus on researching wearables like smartwatches to assess sleep duration, smartphones offer a more scalable potential. Nearly 90% of the US population uses smartphones, but only 20% use smartwatches or fitness trackers^7^. In addition, the population of wearable users consists mostly of wealthy, educated, and white demographic groups. This calls into question whether wearables can equitably reach those with the highest health needs^8^. To make matters worse, in studies using wearables, most users abandon them after only a few weeks of use^9^. These results stand in contrast to smartphone usage data, where the most disadvantaged people may even be smartphone-dependent, causing rising concern about overuse.

Therefore, while appreciating research on wearables, we instead focus on what information smartphones can offer. Our 2019 review of smartphones for sleep in mental health noted that current apps monitoring sleep mostly focus on self-reported data, such as logging hours^10,11^. However, we identify new methods, like digital phenotyping, as opportunities to better capture sleep duration. Digital phenotyping is defined as the “moment-by-moment quantification of the individual-level human phenotype in situ from personal digital devices” (one relies only on smartphones people already own and use today.

While researchers have already made impressive findings on digital phenotyping and sleep, much of it relies on actigraphy work that does not utilize smartphones^12^ or wearables like Fitbits^13^. Although studies using smartphones to monitor sleep show, this approach to be feasible^14^results have been challenging to replicate because study apps are either proprietary or sometimes no longer even available^15^. To correct these errors, and to showcase the feasibility and effectiveness of using smartphones to monitor sleep, we used the open-source mindLAMP app to create and assess smartphone-based sleep measures.

In this study, we analyzed a cohort of 67 technology-enabled participants who completed various sleep-monitoring surveys and mental health questionnaires through the mindLAMP app over the course of 28 days. These participants also collected smartphone sensor data, including device usage, geolocation, and accelerometer data. We used this passively collected sensor data to create daily sleep duration estimates. We compared these estimates to survey results, and later created a predictive model to digitally phenotype sleep habits. These participants were recruited for a separate study involving the digital phenotyping of college students, but in this analysis, we focus only on sleep. In this paper, we show how sleep duration, as estimated by smartphones, proves to be a highly valuable metric in the process of digital phenotyping.

## Methods

Sleep data for this analysis was gathered from a prior study which is outlined in a protocol paper accessible in JMIR Protocols, but has never been reported on before^16^. We summarize the protocol below. This study was conducted in accordance with the Declaration of Helsinki and was reviewed by the IRB / Committee of Clinical Investigation of Beth Israel Deaconess Medical Center.

### Study

Data for this study was collected using mindLAMP, an open-source app developed by the Digital Psychiatry lab at Beth Israel Deaconess Medical Center^17^. Participants were recruited using social media. After enrollment, participants were given daily activities (such as mindfulness or gratitude journaling), daily surveys (daily sleep duration, daily sleep quality), and weekly surveys (Patient Health Questionnaire-9 (PHQ-9), Generalized Anxiety Disorder-7 (GAD-7), Perceived Stress Scale (PSS), UCLA Loneliness Survey, Pittsburgh Sleep Quality Index (PSQI), Digital Working Alliance Inventory (DWAI), and TAM-related questions). Patients enrolled completed an informed consent quiz detailing the expectations of this study and, if they passed, also completed written informed consent.

### Enrollment

Sixty-seven participants were used for this analysis. Of the 108 participants that entered the enrollment period of the study, 34 were discontinued after not completing any activities in the app for 5 consecutive days. Seventy-four participants completed the study, but seven participants were excluded from the analysis due to participation in a previous iteration of this study.

### Passive sleep duration estimates

Smartphone sensor data was used to estimate time spent in bed as a proxy for time spent sleeping. Sensor data collected includes an accelerometer and device usage data. When a participant turns on their phone, mindLAMP reports usage data indicating the status of the phone (on and unlocked, on and locked, off and unlocked, off and locked.) Accelerometer data was reported as a three-dimensional vector broken into x, y, and z cartesian components in units of g (9.81 m/s/s). From the accelerometer, we computed jerk, the first derivative of acceleration. When in use, phones report high magnitudes of jerk vectors, suggesting an active state. To determine the threshold in jerk magnitude, which distinguishes between activity and inactivity, we employed Otsu’s method^18^. The original application of Otsu’s method involves image processing. Otsu’s method categorizes pixels from images into foreground and background by determining the threshold, which minimizes the sum of within-group variances^19^. Whether the gray level of pixels falls above or below this threshold determines whether it belongs to the foreground or background. In a manner similar to image processing, we instead apply Otsu’s method to categorize accelerometer jerk values into high and low states. Participants were assumed to be active and awake when either accelerometer jerk magnitudes were above the jerk threshold, or device use data reported an on-event. Other bouts of inactivity were assumed to be sleep periods. Periods of missing data were concatenated according to their state. For matching states separated by missing data (e.g., asleep state -> missingness -> asleep state), missing periods were assumed to be the same as the two matching states. For mismatches (e.g., asleep state -> missingness -> awake state), participants were assumed to be inactive, because a resumption in data collection would most likely be caused by user activity and therefore indicates a transition from an inactive to an active state.

### Mixed linear regression

For this study, all regression coefficients were calculated using the statsmodels package in python 3.8. Because each participant may report different baseline PSQI scores, we used a mixed linear model, where we considered the participant to which data belongs to be a random effect. Therefore, the intercept was random. We considered the relationships between each variable and PSQI to be fixed effects.

### Predictive model

To evaluate whether PSQI can be predicted from available data, we constructed a simple linear model using active and passive data as predictor variables. This model was created in python 3.8 using the scikit-learn package. However, PSQI, daily surveys, and passive sleep duration estimates were collected on different schedules. PSQI was administered weekly, whereas sleep duration, sleep quality, and sleep duration estimates were collected daily. Patient-reported PSQI was intended to reflect perceived sleep duration over the course of the previous week. Therefore, for each reported value of PSQI, we averaged survey scores and sleep duration estimates between the timestamp when each PSQI was taken and the timestamp when the PSQI was taken previously. This resulted in congruent shapes in data between all predictor variables and the target variable. This model was validated and errors were reported using leave-one-out cross-validation (LOOCV).

### Reporting summary

Further information on research design is available in the Nature Research Reporting Summary linked to this article.

## Results

### Demographics and data quality

Recruited participants were given access to the open-source mindLAMP mobile application. Using mindLAMP, these participants completed daily and weekly surveys. Of these participants, 67 successfully completed surveys required for sleep analysis (weekly PSQI, daily sleep duration, daily sleep quality). The demographic information of these participants can be seen in Table 1. Participants had a mean age of 20.0 with a standard deviation of 2.0. Participants were primarily female (65.7%), with a slight majority of patients identifying as white (56.7%).

**Table 1:** Demographic information of participants passing enrollment and screening criteria.

Characteristics	*n*	%
Gender		
Female	44	65.7
Male	15	22.4
Non-binary	7	10.4
No response	1	1.5
Race/ethnicity		
American Indian/Alaskan	0	0
Asian	19	28.4
African American	2	2.9
Latino or Latina	8	11.9
White	38	56.7

The protocol prompted participants to provide 28 days’ worth of survey data, with one of each daily survey every day and one of each weekly survey taken every week. However, due to user error, such as responding to a daily survey twice in a single day, participants at times deviated from this protocol. The sleep-monitoring surveys of interest in this study were daily sleep duration, daily sleep quality, and weekly PSQI. Participants provided an average of 28.9 (standard deviation of 5.3) daily surveys and an average of 4.6 (standard deviation of 1.1) weekly PSQI surveys. Phone sensor data (“passive data”), including accelerometer and screen use data, were collected from the moment each participant enabled data collection to the moment data collection was disabled at the end of the study. However, only passive data collected during the study period was included in the analysis. The collection of phone sensor data allows for the computation of many secondary metrics, including time spent at home, time spent using cell phones, and others. However, for the purposes of this paper, we will focus only on using an accelerometer and screen use data to estimate sleep duration (“passive sleep”).

In order to accurately estimate sleep duration from passive data, participants had to meet a minimum level of data coverage. For this analysis, all accelerometer data for each participant was split into 24-h intervals. Per-interval data coverage was calculated by determining the number of 5-second bins containing at least one data point. 24-h intervals with a data coverage of less than 60% were excluded. After imposing these conditions, 65 participants remained with at least one 24-h period of applicable data, with an average of 19.2 (standard deviation 5.7) of these periods per participant. Using these 65 participants, sleep duration was estimated on a per-night basis.

### Sleep correlations

Participants answered a variety of surveys (“active data”) for this study in addition to the sleep-monitoring surveys. For the purposes of this analysis, we considered a number of surveys that may reasonably be associated in some way with sleep habits. These included the following: General Anxiety Disorder-7 (GAD-7), Perceived Stress Scale (PSS), Patient Health Questionnaire-9 (PHQ-9), Prodromal Questionnaire-16 (PQ-16), and the Pittsburgh Sleep Quality Index (PSQI). These surveys were conducted on a weekly basis. As mentioned, we administered a daily survey asking participants to report time spent asleep the previous night (“active sleep duration”) and another daily survey to report trouble sleeping on a scale of 0-10, with higher scores indicating lower quality (“active sleep quality”), again on the previous night. We give survey administration details in our associated protocol paper.

**Fig. 1:**
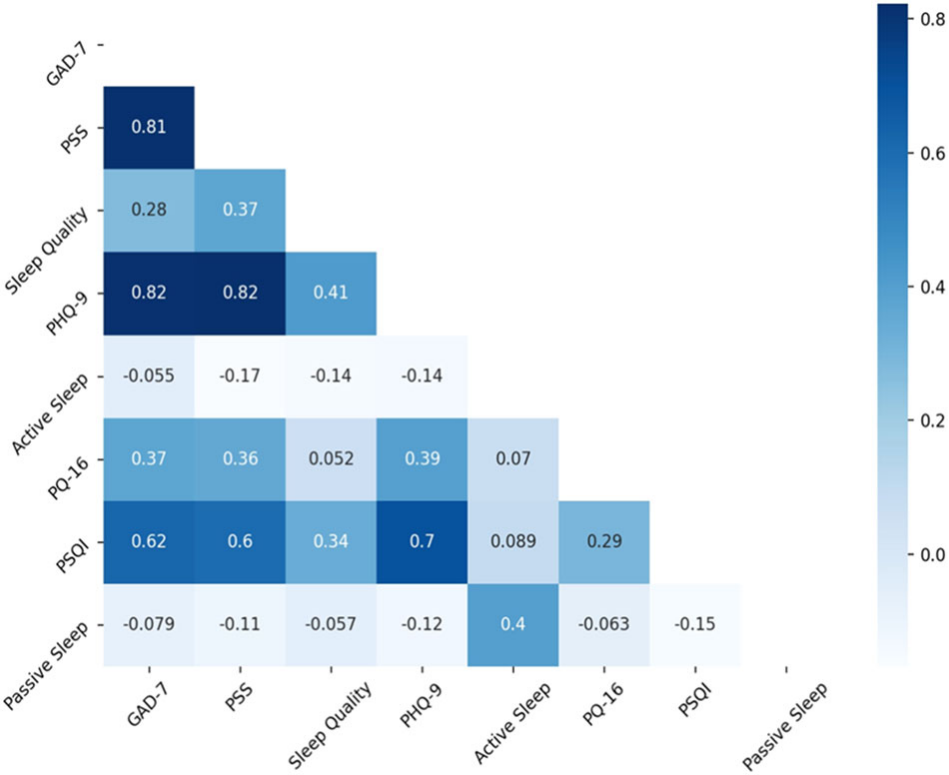
Correlation matrix. Correlation matrix between mean metrics. Values reported in the table are Pearson correlation coefficients. [color].

Survey responses were mapped to integer values and averaged over the course of the study for each participant. In addition, per-day average sleep duration estimates were computed for each participant. Correlations, reported as Pearson coefficients, between each data stream were plotted against each other (Fig. 1). Mean sleep duration as estimated by passive data correlates with mean sleep duration as reported by daily surveys (*r* = 0.39, *p* < 0.05). Although active data and passive data were collected over the same study period, neither passive data nor active data were necessarily available every night over the period. This introduced a discrepancy where some days included passive data without active data and vice versa. Therefore, we also computed correlations between survey-reported sleep duration and passive sleep duration estimates, including only those nights containing pairs (i.e., days in which both active and passive data sleep duration estimates are available). After this change, the correlation between the two data streams was higher (*r* = 0.83) and significant (*p* < 0.05).

### Relationship between passive data, active data, and PSQI

**Fig. 2:**
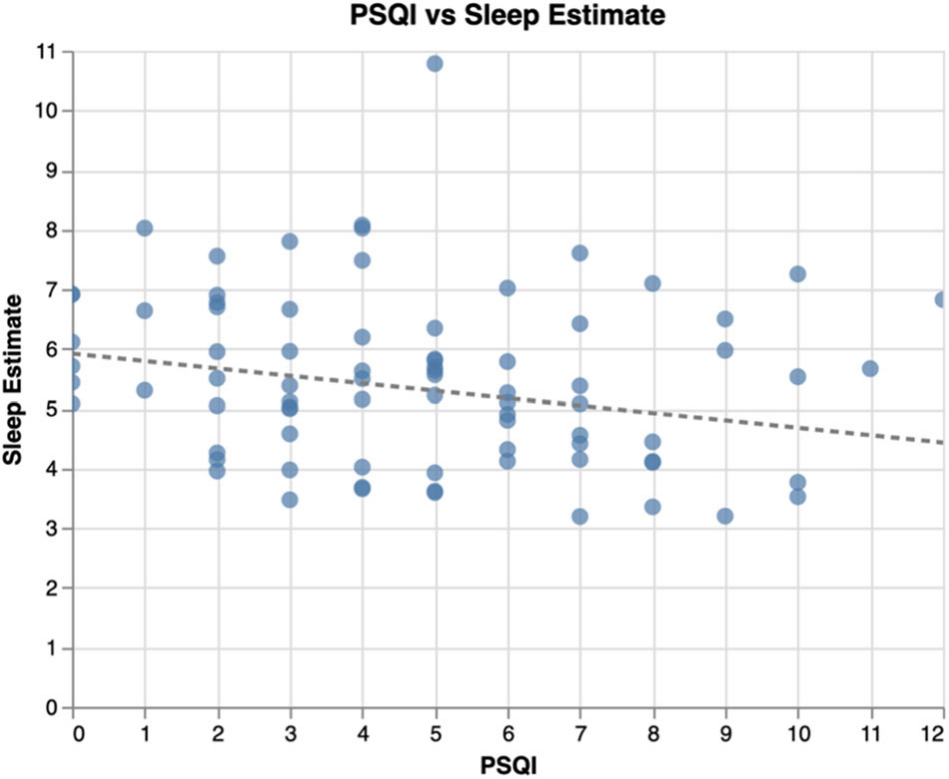
Estimated sleep duration vs weekly survey-reported sleep duration. Estimated sleep duration is derived from mindLAMP and presented in hours on the vertical axis. The survey reported sleep duration is from the PSQI and presented on the horizontal axis in hours.

Without adjusting for other variables, out of both active and passive data, on a week-by-week basis, passive sleep duration estimates were found to be negatively correlated (*r* = −0.24, *p* < 0.05) with PSQI and active sleep quality was found to be positively correlated (*r* = 0.25, *p* < 0.05) with PSQI. This is not surprising, as higher daily sleep quality scores and higher PSQI scores are both indicative of lower sleep quality. We plotted passive sleep duration estimates against weekly PSQI across all participants (Fig. 2). Interestingly, the survey reported daily sleep duration (*p* = 0.41) was not found to be correlated with weekly PSQI.

### Mixed model regression

To further ascertain the relationships between variables while accounting for within-subject correlations, we also performed a linear regression of PSQI using a mixed linear model. We considered the participant to which data belongs to be a random effect. As such, we fit the slopes between PSQI and each predictor variable as fixed effects with the intercept as a random effect. Predictor variables include initial PSQI, survey scores, and passive sleep duration estimates. Regression coefficients and *p* values for this model were compiled (Table 2).

**Table 2:** Summary of results of mixed model regression to predict weekly PSQI scores.

Variable	Coefficient	St. Error	*p* value
Intercept	2.8	2.0	0.17
Active sleep quality	0.21	0.16	0.19
Active sleep duration	0.40	0.23	0.09
Sleep duration estimate	−0.31	0.14	0.03

Out of these results, passive sleep duration estimates were the most statistically significant factor in predicting PSQI. Interestingly, only passive sleep duration estimates, and not survey-reported sleep duration, yielded a negative coefficient. As expected, survey-reported sleep quality displayed a positive coefficient, but this result was not significant (higher survey-reported sleep quality scores indicate lower quality of sleep).

### Predictive model

**Fig. 3:**
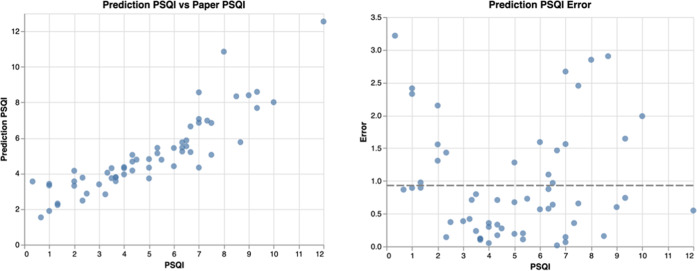
Linear model results. (left) Predicted PSQI(0-14) vs reported PSQI. (right) error between predicted PSQI and reported PSQI for every predicted and reported value (mean error in dashed gray).

The PSQI encompasses components of both sleep quality and duration. This raises the question of whether some combination of daily sleep quality surveys, daily sleep duration surveys, and passive sleep duration estimates can predict PSQI. We created a simple linear predictive model, as described in the methods section of this paper, to determine whether PSQI can be predicted by the same data streams. This model was validated using leave-one-out cross-validation. Model results and prediction errors were plotted (Fig. 3). Mean absolute error across all predicted data were 0.93, suggesting this model predicts PSQI to within a point on average. PSQI itself ranges from 0 to 14.

## Discussion

Our study demonstrates the potential of using smartphone-based digital phenotyping to capture scalable and actionable data that can advance both care and research. This analysis of 67 college students showed how mean passive sleep duration estimates correlate (*r* = 0.83) with mean sleep duration reported by daily surveys. In our mixed model, we found that daily sleep duration estimated by passive data was most strongly associated with a decrease in PSQI in comparison to daily sleep duration surveys. Additionally, our simple linear predictive model gave a mean absolute error of less than 1, indicating that passive data streams and active data streams together can accurately predict PSQI.

Our results around estimated sleep duration highlight the utility of this smartphone-based approach to digital phenotyping. The correlation between phone-based sleep duration estimates and self-reported sleep duration rose to a high degree (*r* = 0.83) for nights when both data streams were available. In fact, sleep duration estimates may even outperform self-reported survey results. Patients may forget to submit surveys, and reported estimates vary. Patients become disengaged with surveys over time^20^. On the contrary, sleep estimates using passive data can be produced automatically.

While these results require additional validation, our sleep duration estimates can offer clinical utility today. Given the well-documented challenges around engagement with sleep logs and wearables, including actigraphy, offering smartphone-based sleep monitoring can provide a simple solution accessible to nearly all patients today with no cost or additional equipment required. Clinicians can easily start clinical conversations around sleep results with a patient while reviewing whether these results match what the patient experiences. Our team currently does this in our outpatient clinical work.

Our results suggest an alternative path to validating passive sleep duration estimates outside of direct comparison to survey-reported sleep duration. In digital phenotyping, the absolute accuracy of computed metrics is not necessarily the primary consideration. Rather, we prioritize the utility of this data in informing actionable metrics around sleep, such as the PSQI. Out of passive sleep duration estimates, surveys, and wearable devices, passive sleep duration estimates provided the best option for digitally phenotyping sleep metrics. Overall, our results suggest sleep duration estimates may be more reliable than surveys in evaluating sleep habits. For example, on a weekly basis, only passive sleep duration, and not survey-reported sleep duration, was found to be correlated with a decrease in PSQI. This result appears surprising, considering mean survey-reported sleep duration was highly correlated with mean passive sleep duration estimates. A possible explanation could be that survey reports are inconsistent on a week-by-week basis but reliable when taking the mean over a greater number of days or weeks.

Prior studies have also used smartphone sensors to estimate sleep duration with varying degrees of success. However, many of these methods make a priori assumptions about sleep and wake periods using environmental cues or calendar entries ^21^). Others analyzed a small sample size^22^. On the contrary, our method takes a data-driven approach, making only the unavoidable assumption that sleep periods and inactive periods are the same. Cuttone et al. employed various Bayesian models with varying assumptions^23–25^. These models were data-driven and had success, but only used device usage data. Collecting only screen state data without accelerometer data ignores periods of activity where the phone remains in an off state, such as when a phone remains off but in a participant’s pocket.

Limitations in this method include the assumption that inactive periods and sleep periods are synonymous. Inconsistent smartphone usage may decrease validity. However, this is a challenge applicable to the greater field of digital phenotyping as a whole; any method using smartphone data to parse behavioral patterns will suffer from inconsistent device use. The passive sleep duration estimates are, therefore, most applicable to populations more likely to use their smartphones consistently, such as college students. To mitigate this error in practice, when using this method for research purposes, participants can be prompted to turn on or move their phones moments before getting into bed. Other limitations include that this analysis compared passive sleep duration estimates to the clinically validated PSQI, but did not compare passive sleep duration estimates to actigraphy or polysomnography. Furthermore, our results should be generalized by replication in other data sets.

In conclusion, our data-driven approach to estimate sleep duration correlates with survey results. This suggests our method may be used to capture changes in sleep habits over time. In the digital phenotyping process, passive sleep duration estimates can be used in isolation or in conjunction with other data streams to model actionable metrics such as the PSQI.

### Supplementary information

Editorial Policy Checklist

Reporting Summary

## Data Availability

The dataset for this analysis contains sensitive patient information and, therefore, cannot be shared publicly. De-identified data may be available upon request.

## Code availability

Code for the open-source mindLAMP app, as well as the data analysis pipeline LAMP-cortex, can be found in the repositories at https://github.com/BIDMCDigitalPsychiatry.
